# Characterization of global research trends and prospects on platinum-resistant ovarian cancer: a bibliometric analysis

**DOI:** 10.3389/fonc.2023.1151871

**Published:** 2023-06-05

**Authors:** Yuanqiong Duan, Peixuan Zhang, Tianyue Zhang, Lu Zhou, Rutie Yin

**Affiliations:** ^1^ Department of Obstetrics and Gynecology, West China Second Hospital of Sichuan University, Chengdu, Sichuan, China; ^2^ Key Laboratory of Birth Defects and Related Diseases of Women and Children, Ministry of Education, Sichuan University, Chengdu, China

**Keywords:** platinum-resistant, ovarian cancer, bibliometrics analysis, Citespace, VOSviewer

## Abstract

**Background:**

In the last decades, growing attention has been focused on identifying effective therapeutic strategies in the orphan clinical setting of women with platinum-resistant ovarian cancer (PROC), generating thousands of original articles. However, the literature involving bibliometric analysis of PROC has not been published yet.

**Objective:**

This study hopes to gain a better understanding of the hot spots and trends in PROC by conducting a bibliometric analysis, as well as identify potential new research directions.

**Methods:**

We searched the Web of Science Core Collection (WOSCC) for PROC-related articles published between 1990 and 2022. CiteSpace 6.1.R2 and VOS viewer 1.6.18.0 were primarily utilized to evaluate the contribution and co-occurrence relationships of various countries and regions, institutes, and journals and to identify research hotspots and promising future trends in this research field.

**Results:**

A total of 3,462 Web of Science publications were retrieved that were published in 671 academic journals by 1135 authors from 844 organizations in 75 countries and regions. The United States was the leading contributor in this field, and the University of Texas MD Anderson Cancer Center was the most productive institution. Gynecologic Oncology was the most productive journal, while the Journal of Clinical Oncology was the most cited and influential. Co-citation cluster labels revealed the characteristics of seven major clusters, including synthetic lethality, salvage treatment, human ovarian-carcinoma cell line, PARP inhibitor resistance, antitumor complexes, folate receptor, and targeting platinum-resistant disease. Keywords and references burst detection indicated that biomarkers, genetic and phenotypic changes, immunotherapy, and targeted therapy were the most recent and most significant aspects of PROC research.

**Conclusion:**

This study conducted a comprehensive review of PROC research using bibliometric and visual techniques. Understanding the immunological landscape of PROC and identifying the population that can benefit from immunotherapy, especially in combination with other therapeutic options (such as chemotherapy and targeted therapy), will continue to be the focal point of research.

## 1 Introduction

Ovarian cancer (OC) is the most lethal tumor of the female reproductive system, with a death-to-incidence ratio that is remarkable. Due to the ambiguity of clinical symptoms and the absence of valid screening methods for OC, more than 70% of patients are already in an advanced stage (FIGO III–IV) at the time of diagnosis (1, 2), with a 5-year overall survival rate of 40–45% (3). Combining cytoreductive surgery and adjuvant chemotherapy is the mainstay of ovarian cancer treatment. The most active therapeutic agents for newly diagnosed advanced ovarian cancer are 6 cycles of platinum-based chemotherapy (4), but 20% of patients still have a poor primary outcome, a response rate of <15% to subsequent chemotherapy, and a median survival time of less than 1 year (5). Although the majority of patients achieve complete remission after initial treatment (i.e., surgery plus chemotherapy), the vast majority of patients (65% to 80%) experience recurrent relapses within the first 5 years (before PARP inhibitors were approved), resulting in platinum resistance.

Patients with platinum-resistant ovarian cancer (PPROC) experience poor oncologic outcomes. The platinum-free interval (PFI) is a vital predictor of treatment efficacy and prognosis (6). PROC is typically defined as progression within six months of completing cytoreductive surgery and platinum-based therapy, which occurs in approximately 20% of patients after first-line platinum-based therapy (7). There are currently no effective treatment options available for these patients. PPROC’s primary treatment is individualized based on the patient’s characteristics, but typically includes cytotoxic therapy and anti-angiogenesis treatment modalities. In the open-label, randomized phase III AURELIA trial, the remission rate of bevacizumab in combination with chemotherapeutic agents was 27.3%, prolonging patients’ PFS by approximately 3 months, but there was no significant improvement in OS at the final analysis [HR 0.85 (95% CI 0.85–1.08); P=0.174; median 16.6 months with bevacizumab plus chemotherapy versus 13.3 months with chemotherapy alone] (8). However, Bamias et al. (9) investigated the effect of bevacizumab use after progression of disease (PD) in patients randomized to chemotherapy alone and concluded that in these exploratory analyses of nonrandomized subgroups, bevacizumab use, either with chemotherapy or after PD on chemotherapy alone, improved OS compared with no bevacizumab. In addition, PARP inhibitors (PAPRi) have demonstrated preliminary efficacy for PPROC. Moore’s study showed a 33% objective response rate (ORR) with niraparib monotherapy in ≥ 3-line platinum-resistant relapsed ovarian cancer and a 43% ORR with niraparib monotherapy in ≥ 4-line platinum-resistant ovarian cancer with BRCA mutations, as well as a benefit for patients regardless of BRCA mutation or homologous recombination deficiency (HRD) status (10). In addition, accumulating evidence demonstrates that mono-chemotherapy agents provide a comparable response to poly-chemotherapy regimens while minimizing the likelihood of adverse events (5, 8, 11). The treatment of PPROC has been an unmet clinical need for the gynecologic oncology community until now.

Over the past few decades, the identification of effective therapeutic strategies in the orphan clinical setting of women with PPROC has generated tens of thousands of original articles. However, in this age of information overload, screening these numerous, disparate, and dispersed publications is particularly time-consuming, laborious, and inefficient. This issue can be effectively resolved with the assistance of bibliometric analysis, which enables us to grasp the hot spots and trends in a particular field swiftly (12). Text-mining of information extracted from research literature (13) is facilitated by bibliometric analysis based on an artificial intelligence-based algorithm. It can not only provide a quantitative and statistical analysis of publications in specific areas (14, 15), but also comprehensively analyze the development of a discipline and intuitively comprehend trends at the frontier by presenting numerous data as knowledge maps (12). Therefore, bibliometric analysis, which has been used to evaluate the interrelationships and effects of published articles and citations, is superior to conventional review in terms of information data processing and visualization (16), as it employs mathematical and statistical techniques to recognize and organize the structure of knowledge.

However, bibliometric research pertaining to PROC has not yet been published. Frontiers in Oncology has served as a platform for publishing bibliometric research in various medical specialties, particularly gynecological oncology, with the goal of expanding the scientific community’s understanding of the methodologies used to advance the field (17). We provide an overview of the research literature from 1990 to 2022 to illustrate the evolution of platinum-resistant OC treatment. The primary purpose of our study was to conduct a bibliometric analysis to quantify and identify the status quo and emerging issues of platinum-resistant ovarian cancer, thereby laying the groundwork for future investigation.

## 2 Materials and methods

### 2.1 Sources of bibliometric data

**Figure 1:**
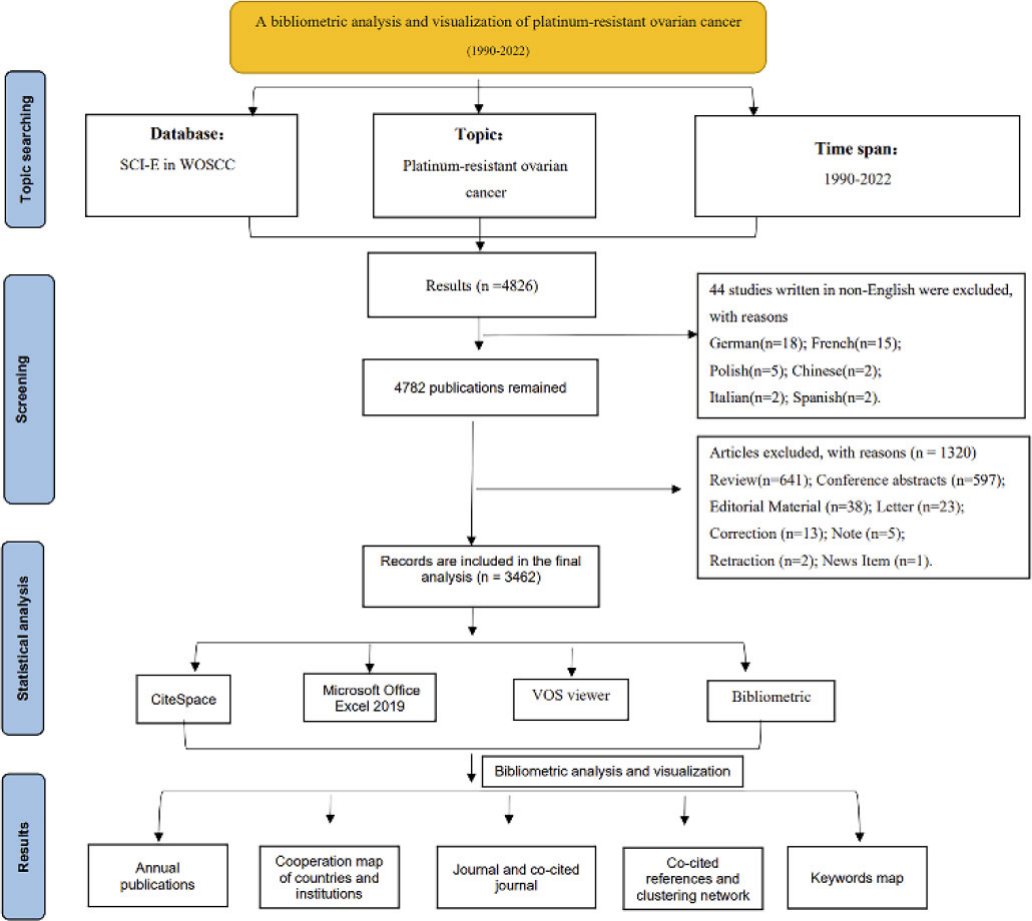
Flow chart of literature inclusion and exclusion.

The Web of Science Core Collection (WOSCC) is a canonical online database providing a standardized and up-to-date reference dataset for scientific research and analysis, among which the Science Citation Index Expanded (SCI-E) is regarded as the most suitable database for bibliometric analysis (14, 18). In this study, literature on platinum-resistant OC was systematically retrieved from the SCI-E of WOSCC. The publication date was unrestricted, while the final retrieval was conducted on December 31, 2022. Principally, OC (Strategy A) and platinum resistance-related (Strategy B) terms were used as descriptors (**Supplemental Table 1**). Finally, a Boolean algorithm consisting of “A AND B” (Strategy C) was employed to ensure that all articles retrieved fell within the field of platinum-resistant OC. Two researchers independently conducted the retrieval. During the retrieval process, disagreements were resolved by consulting a third colleague or the entire academic team. To ensure proper interpretation of the results, English was the only language of publication. In terms of document types, only articles were included in the study, while all others were excluded. This study was designed according to the PRISMA recommendations. The flow diagram is depicted in **Figure 1**.

### 2.2 Analysis methods and visualization

Bibliometric analysis is an emerging tool for rapidly exploring the structures and trends of a subject or domain using statistical methods and visualization to identify relevant nodes and extract useful information from large information volumes (18). CiteSpace, VOS Viewer, SCI2, NetDraw, and HistCite are currently the most commonly used bibliometric software programs (19). In this study, the bibliometrics software CiteSpace (6.1.R2) (Chaomei Chen, Drexel University, USA) and VOS viewer (1.6.18.0) were primarily used for data processing and result visualization due to their respective features and benefits.

CiteSpace is a free Java application that can visualize and analyze scientific literature trends and patterns. It was developed by Professor Chaomei Chen for progressive knowledge domain visualization and the identification of critical points in the evolution of a field, especially intellectual turning points and junctures (20). In addition, this analysis tool is effective at revealing cooperation, key points, internal structure, potential trends, and dynamics in a certain field (12). Therefore, CiteSpace (6.1.R2) was utilized to analyze and visualize the co-occurrence of countries/regions and institutions, a dual-map of journals, trends of high-frequency keywords, co-cited references, and citation bursts for references.

The VOS viewer was developed by Leiden University’s Center for Science and Technology Studies and is used to visualize science mapping. VOS viewer is software that is proficient at creating, visualizing, and navigating network-data-based maps (21). We identified productive journals, co-cited journals, and related knowledge maps using VOS viewer (1.6.18.0) and bibliographic data.

Microsoft Office Excel 2019 was also used to manage the database and analyze the annual publications. In addition, we extracted the 2021 impact factor (IF), H-index, and JCR division of journals from the Web of Science’s Incites Journal Citation Reports and bibliometric (22) (an R-tool for comprehensive science mapping analysis) (https://www.bibliometrix.org/home/).

### 2.3 Patient and public involvement

No patients were involved.

## 3 Results

### 3.1 Annual publication

**Figure 2:**
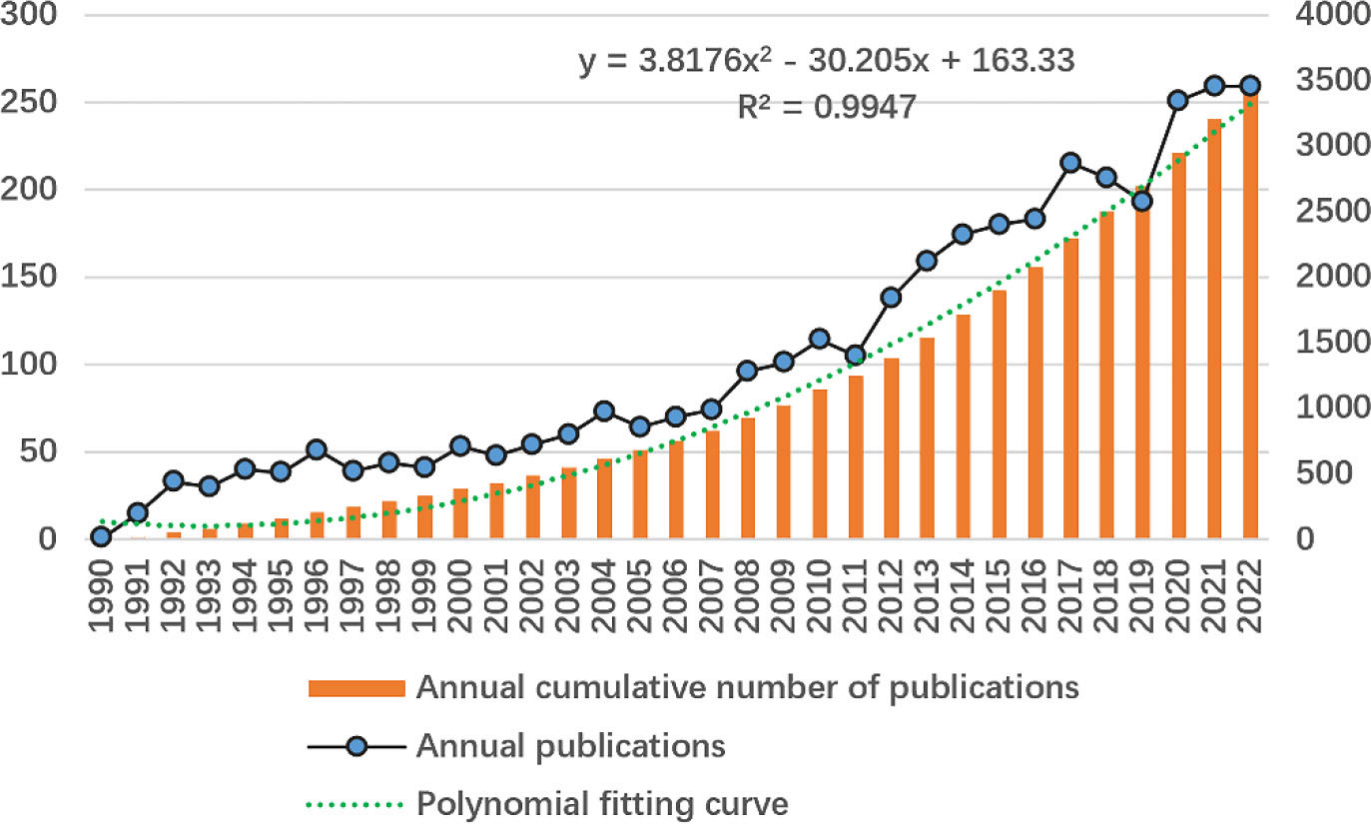
Annual frequency of publications in the field of platinum-resistant ovarian cancer.

According to the data collection strategy, 3,462 unique articles published between 1990 and 2022 were collected. The frequency distribution of the selected articles by publication year is depicted in **Figure 2**. There have been two distinct phases in the development of published articles on platinum-resistant OC: a slow but steady phase from 1990 to 2012, and a phase of rapid growth from 2013 to 2022.

### 3.2 Country/region and institute cooperation map

#### 3.2.1 Country contributions

**Figure 3:**
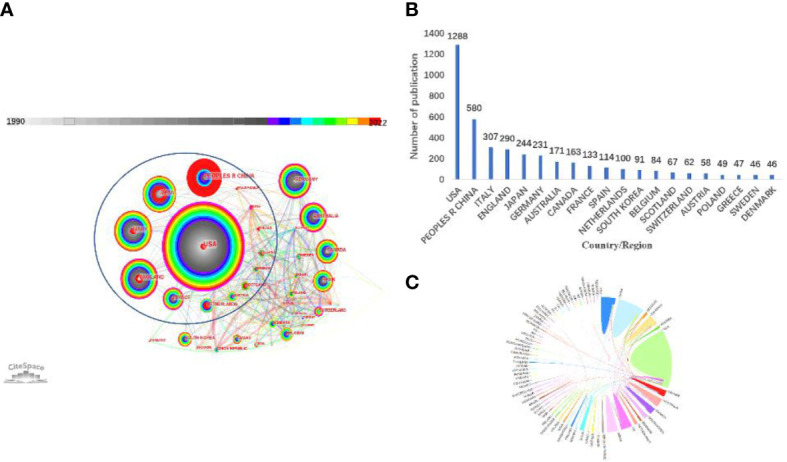
Cooperation map of country/region **(A)** Country/region co-occurrence network. (75 nodes and 385 connection lines emerged. Node and line size represent the number of publications from a country/region and the cooperative relationship in the country/region, respectively. Red nodes indicate that publications of the country/region have a citation or frequency burst. Connecting lines of different colors represent different years. Blue ellipse indicates a burst in the citation/frequency of country/region.) **(B)** Number of publications for top 20 countries/regions. **(C)** The cooperation relationships of countries or regions.

There are 75 countries that have published research on platinum-resistant OC. **Figures 3A, B** characterize the network map and distribution of various countries generated by CiteSpace. The size of each node represents the total number of publications produced by each author, institution, or country (23, 24), whereas the lines between nodes represent citations. In **Figure 3A**, the United States, Germany, England, France, Australia, Russia, and Switzerland are shaded purple due to their high betweenness centrality, which is commonly regarded as a significant turning point that may lead to transformative discoveries and serve as a bridge. The top five countries or regions in terms of centrality were Germany (centrality = 0.22), England (centrality = 0.20), Australia (centrality = 0.19), the USA (centrality = 0.16), and Russia (centrality = 0.13). In **Figure 3B**, The United States of America (USA) was the most productive nation with 1288 published articles (38.2% of the total), followed by China (n = 580), Italy (n = 307), England (n = 290), Japan (n = 244), Germany (n = 231), Australia (n = 171), Canada (n = 163), France (n = 133), and Spain (n = 114). As shown in **Figure 3C**, international collaboration network analysis revealed that USA and United Kingdom collaborated most frequently, followed by USA and China.

#### 3.2.2 Contributions made by the Institute

**Figure 4:**
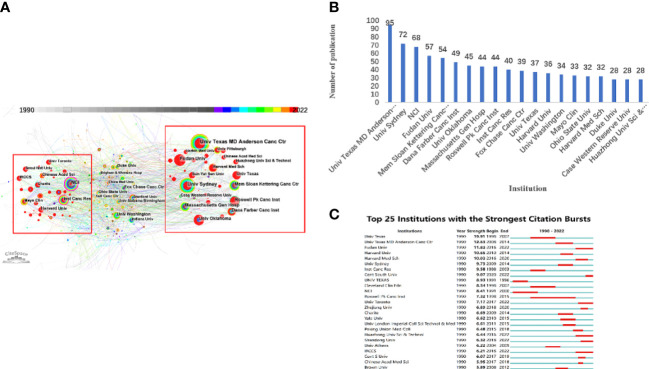
Cooperation map of Institute. **(A)** Institute co-occurrence network. (844 nodes and 1670 connection lines emerged. Node and line size represent the number of publications from an institute and the cooperative relationship in institute, respectively. Red nodes indicate that publications of the country/region have a citation or frequency burst. Connecting lines of different colors represent different years. Red rectangles show a burst in the citation/frequency of institute.) **(B)** Number of publications for top 20 Institutes. **(C)** Top 25 Institutes with the Strongest Citation Bursts. (Blue bars and red bars mean that some keywords are cited frequently in a certain period).

Globally, 844 institutions, independently or in collaboration, have published PROC-related research. **Figures 4A, B** portray the network map and distribution of different organizations yielded by CiteSpace. There are seven organizations among the top 10 institutes affiliated with the United States from an institutional standpoint, with the University of Texas MD Anderson Cancer Center ranking first. This ranking reflects the United States’ superior scientific prowess. In **Figure 4B**, the University of Texas MD Anderson Cancer Center was the most productive institution with 95 papers in the field of PROC (2.8% of the total), followed by the University of Sydney (72 papers, 2.1%), the National Cancer Institute (68 papers, 2.0%), Fudan University (57 papers, 1.7%), and the Memorial Sloan Kettering Cancer Center (54 papers, 1.6%). **Figure 4C** illustrates the top 25 institutions with the strongest citations or frequency bursts.

### 3.3 Journal and co-cited journal

Using VOS viewer, we conducted a co-citation and co-cited journal analysis to identify the most active and influential journals in the PROC field. The leading ten journals in the field of PROC are listed in **Table 1**. It is observed that Gynecologic Oncology (448, 13.28%) ranks first, followed by the Journal of Clinical Oncology (267, 7.92%), the International Journal of Gynecology (207, 6.14%), the Annals of Oncology (135, 4.00%), and Clinical Cancer Research (131, 3.88%). Of the top 10 journals, 5 are from the US, 3 from England, and 2 from the Netherlands. In addition, there are 4 journals in the top 10 with impact factors >10 and H-Index > 200. These include Annals of Oncology (IF: 51.769; H-Index: 210), Journal of Clinical Oncology (IF: 50.717; H-Index: 494), Clinical Cancer Research (IF: 13.801; H-Index: 282), and Cancer Research (IF: 13.312; H-Index: 411). Among the top 10 journals, Anticancer Research has the lowest impact factor (IF: 2.435; H-Index: 110). Based on the cited number of publications, the Journal of Clinical Oncology (13292) ranks first, followed by Gynecologic Oncology (10064), Cancer Research (9996), Clinical Cancer Research (6189) and British Journal of Cancer (3659), as shown in **Table 2**. There are 7 journals with impact factors >10, H-Index > 200, including New England Journal of Medicine (176.079;933), Nature (69.504;1096), Annals of Oncology (51.769;210), Journal of Clinical Oncology (50.717;494), Clinical Cancer Research (13.801;282), Cancer Research (13.312;411), and Proceedings of the National Academy of Sciences of the United States of America (12.779;699).

**Table 1:** The top 10 journals of platinum-resistant ovarian cancer research.

Rank	Journal	N (%)	Citations	IF (2021)	H-Index (2021)	JCR division	Country
1	Gynecologic Oncology	448(13.28%)	13139	5.304	147	Q2	USA
2	Journal of Clinical Oncology	267(7.92%)	11915	50.717	494	Q1	USA
3	International Journal of Gynecology & Obstetrics	207(6.14%)	2253	4.447	88	Q3	Netherlands
4	Annals of oncology	135(4.00%)	3024	51.769	210	Q1	England
5	Clinical Cancer Research	131(3.88%)	7918	13.801	282	Q1	USA
6	Cancer Research	131(3.88%)	8162	13.312	411	Q1	USA
7	Anticancer Research	93(2.76%)	1629	2.435	110	Q4	Greece
8	Cancers	87(2.58%)	796	6.575	53	Q2	USA
9	British journal of Cancer	83(2.46%)	4131	9.075	212	Q1	England
10	Cancer chemotherapy and pharmacology	67(1.99%)	2097	3.288	100	Q3	USA

N (%): number of publications; % of total publications.

**Table 2:** The top 10 cited journals of platinum-resistant ovarian cancer research.

Rank	Journal	Cited Number	IF (2021)	H-Index (2021)	JCR division	Country
1	Journal of Clinical Oncology	13292	50.717	494	Q1	USA
2	Gynecologic Oncology	10064	5.304	147	Q2	USA
3	Cancer Research	9996	13.312	411	Q1	USA
4	Clinical Cancer Research	6189	13.801	282	Q1	USA
5	British journal of Cancer	3659	9.075	212	Q1	England
6	New England journal of Medicine	3651	176.079	933	Q1	USA
7	Annals of oncology	3633	51.769	210	Q1	England
8	Nature	3230	69.504	1096	Q1	England
9	Proceedings of the National Academy of Sciences of the United States of America	3125	12.779	699	Q1	USA
10	Oncogene	2926	8.756	312	Q1	England

**Figure 5:**
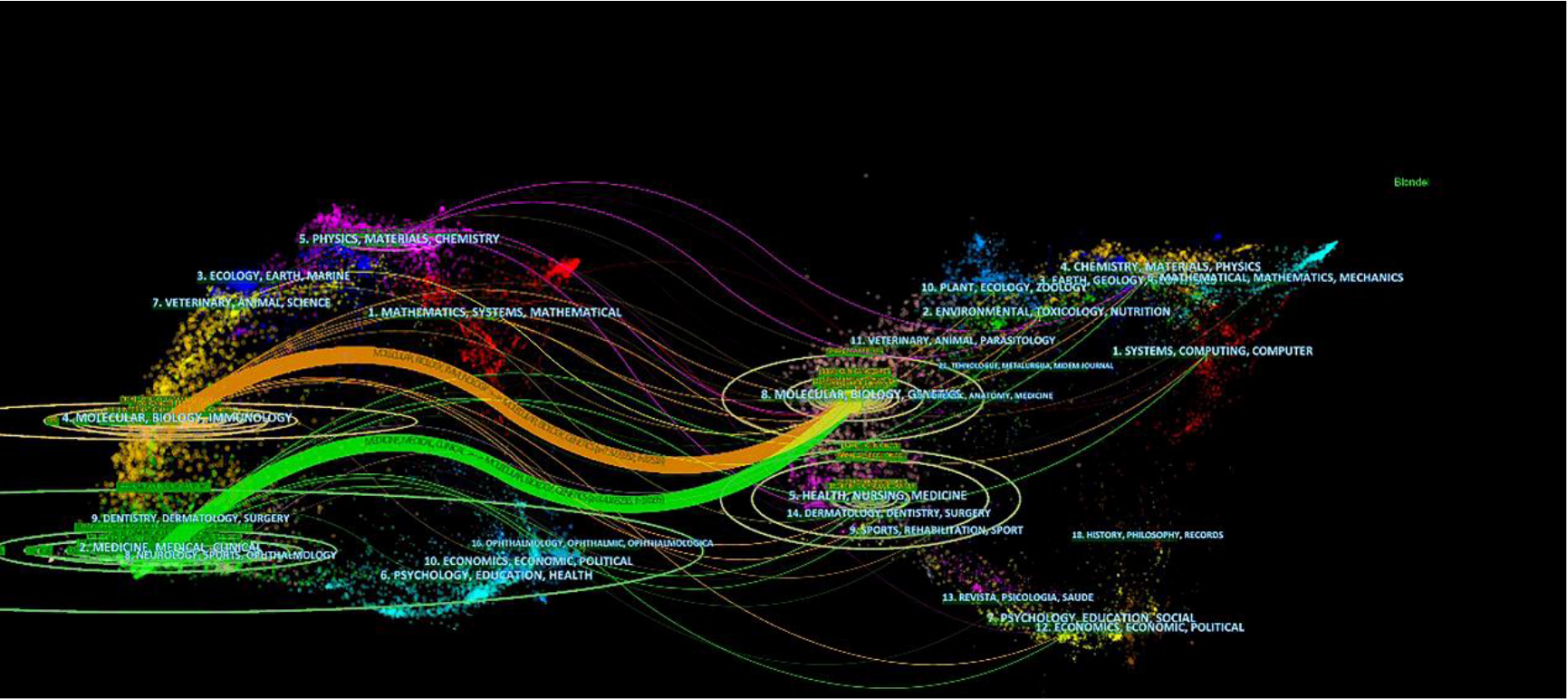
The dual-map overlay of journals related to platinum-resistant ovarian cancer research. (Notes: The citing journals were at left, the cited journals were on the right, and the colored path represents citation relationship. In the citing map, the more papers the journal publishes, the longer the vertical axis of the ellipse, and the greater the number of authors, the longer the horizontal axis of the ellipse.).

Furthermore, as illustrated in **Figure 5**, a dual-map overlay of the journals was created to analyze the relationship between subject categories and platinum-resistant OC. The citation relationships are displayed as spline waves rendered primarily in green, orange, purple, and yellow. The spline curves extend from the leftmost citing journals to the rightmost cited journals. These interactions illustrate the flow and interconnections between various areas (25). Identified are the two primary citation paths, colored orange and green. The orange citation path indicates that the majority of studies published in molecular, biology, and immunology journals were cited by studies published in molecular, biology, and genetics journals. In addition, the green bar indicates that the majority of citations to studies published in medicine, medical, and clinical journals came from studies published in molecular, biological, and genetics journals.

### 3.4 Co-cited references and clustering network

Co-cited references are those that have been cited by multiple publications. Nonetheless, the knowledge base is the collection of co-cited references cited by the relevant research community (20, 26), which is not identical to highly cited references. We performed a co-citation analysis using CiteSpace on the 3,462 articles that were retrieved. **Table 3** presents the ten references that are co-cited the most frequently in PROC studies. The most commonly quoted citation was “Bevacizumab combined with chemotherapy for platinum-resistant recurrent ovarian cancer: The AURELIA open-label randomized phase III trial,” published by Pujade-Lauraine E. Additionally, other significant co-cited references are primarily addressed with “integrated genomic analyses of ovarian carcinoma”, “Molecular mechanisms of cisplatin resistance”, and “Ovarian cancer statistics”. These studies have promoted the evolution of the field by improving the prognosis of platinum-resistant ovarian carcinoma to some extent.

**Table 3:** The top 10 co-cited references related to platinum-resistant ovarian cancer research.

Rank	Citation Counts	The title of Article	Year	Journal			
				Name	Country	IF (2021)	H-Index (2021)
1	115	Bevacizumab combined with chemotherapy for platinum-resistant recurrent ovarian cancer: The AURELIA open-label randomized phase III trial	2014	Journal of Clinical Oncology	USA	50.717	494
2	104	Integrated genomic analyses of ovarian carcinoma	2011	Nature	USA	69.504	1096
3	88	Cancer statistics, 2014	2014	CA-A Cancer Journal for Clinicians	USA	286.13	144
4	78	Global cancer statistics 2018: GLOBOCAN estimates of incidence and mortality worldwide for 36 cancers in 185 countries	2018	CA-A Cancer Journal for Clinicians	USA	286.13	144
5	76	Whole-genome characterization of chemo resistant ovarian cancer	2015	Nature	England	69.504	1096
6	72	Global cancer statistics	2011	CA-A Cancer Journal for Clinicians	USA	286.13	144
7	71	Niraparib Maintenance Therapy in Platinum-Sensitive, Recurrent Ovarian Cancer	2016	New England journal of Medicine	USA	176.079	933
8	69	Cancer Statistics, 2017	2017	CA-A Cancer Journal for Clinicians	USA	286.13	144
9	67	Molecular mechanisms of cisplatin resistance	2012	Oncogene	England	8.756	312
10	65	Ovarian cancer statistics, 2018	2018	CA-A Cancer Journal for Clinicians	USA	286.13	144

**Figure 6:**
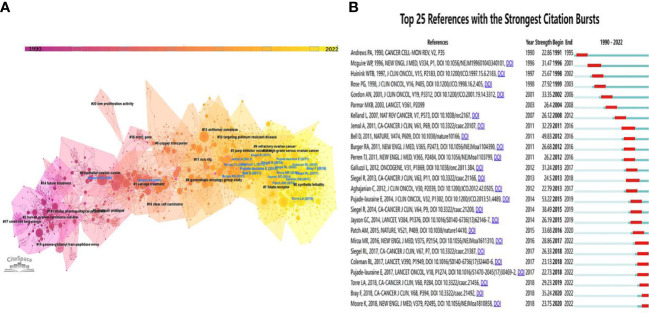
**(A)** Cluster view of co-cited references in platinum-resistant OC research. **(B)** Top 25 co-cited references with the Strongest Citation Bursts. (Blue bars and red bars mean that some keywords are cited frequently in a certain period).

Since the cluster nomenclature reflects the study frontiers in a particular field, we took the additional step of clustering the co-cited references using the CiteSpace software to generate a graphical co-citation map. Clustering analysis was used to divide the retrieved original articles into 21 clusters, as shown in **Figure 6A**. Notably, the modularity Q score is 0.8091 while the mean silhouette value is 0.9147, indicating that the clustering structure is stable and highly persuasive. The literature in each cluster is observed to be closely related and coordinated within a particular field. The cluster labeled “synthetic lethality” (cluster #0) was the largest, followed by “salvage treatment” (cluster #1), “human ovarian-carcinoma cell-line” (cluster #2), and “PARP inhibitor resistance” (cluster #3). In addition, a number of other significant clusters, such as “antitumor complexes,” “targeting platinum-resistant disease,” “folate receptor,” and “targeting platinum-resistant disease,” could represent a shift or breakthrough in this field.

As illustrated in **Figure 6B**, we also examined the top 25 co-cited references with strong citation bursts in ascending order by the starting year of the burst. References with citation bursts are those that are frequently cited over time. The reference with the highest citation strength (strength = 53.22) (8) was also the most cited, followed by Bell D., 2011 (strength = 49.83) (27), Siegel R., 2014 (strength = 38.49) (28), and Bray F., 2018 (strength = 35.24) (29).

### 3.5 Key topics of research hotpots

According to the theory of bibliometrics, keywords indicate hotspots and trends in a research field (30). Keywords, as an important component, can convey the central concept of an academic article (31). Most articles on the same topic or subject area will share one or more keywords, and we can do a deep analysis of the articles by looking at the topological properties and evolution of the co-keyword networks and keyword co-occurrence networks.

#### 3.5.1 Co-occurrence and cluster analysis of keywords

**Figure 7:**
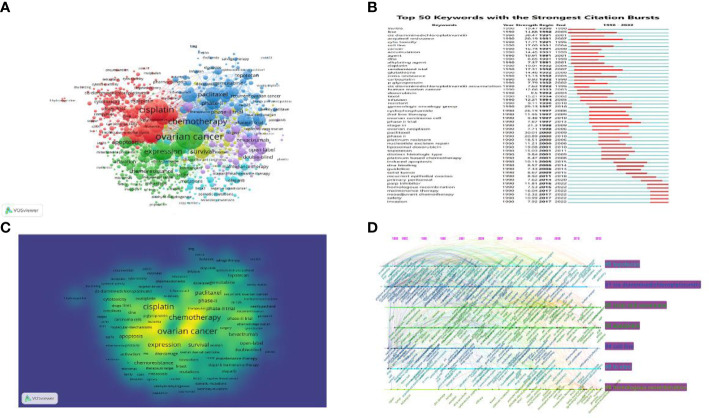
Keywords Map **(A)** Keywords co-occurrence network and clusters in platinum-resistant research (n≥10, relevance score ≥0.081, contains 641 keywords, 6 clusters and 42107 links.) Notes: The size of node and word reflects the co-occurrence frequencies, the link indicate the co-occurrence relationship, and the same color of node represent the same cluster. **(B)** The density map of keywords in PROC research. Notes: The size of word, the size of round, and the opacity of yellow is positively related to the co-cited frequency. It can find the high-frequency co-occurrence terms, which reveal the hotspots in a specific research field. **(C)** Top 50 keywords with the Strongest Citation Bursts. Notes: Blue bars and red bars mean that some keywords are cited frequently in a certain period. **(D)** The timeline view of co-citation clusters. Notes: Visualization of the keyword’s co-citation network time map (1990–2022). In this time map visualization, for each cluster, nodes are organized by their year of publication on horizontal lines. The 2019–2022-time frame permits identifying the latest and most active co-cited clusters.

The VOS viewer excels at creating, visualizing, and exploring keyword co-occurrence maps (21), and we utilized it in PROC research to display not only the keyword co-occurrence network and clusters but also the keyword density map (**Figures 7A, B**). From the title and author keywords of all 3,462 publications, 10430 keywords were extracted, of which 641 occurred more than 10 times. Cluster analysis can reveal the structure of the field’s knowledge (19). Based on the link strength of the co-occurrence terms, the network was divided into the six clusters that can be seen in **Figure 7A**. Cluster 1 (red) contains 180 co-occurrence keywords, including cisplatin, cytotoxicity, drug resistance, *in vitro*, pharmacokinetics, glutathione, p-glycoprotein, and melphalan, among others. The mechanism of cisplatin is the subject of Cluster1. Cluster 2 (green) is primarily associated with the ovarian cancer mechanism, which includes ovarian cancer, gene expression, apoptosis, stem cells, chemoresistance, AKT, MTOR, signaling pathways, aldehyde dehydrogenase, metabolism, etc. Chemotherapy is the subject of Cluster 3 (blue), which contains 109 terms, such as single-agent or weekly paclitaxel, carboplatin, docetaxel, topotecan, clinical-trial, salvage therapy, doxorubicin, etc. Cluster 4 (yellow) contains 74 terms primarily associated with targeted therapy, including bevacizumab, pazopanib, growth factor receptor, vascular endothelial growth factor (VEGF), tyrosine kinase inhibitor, trastuzumab, PD1/PD-L1, pembrolizumab, immune checkpoint inhibitor, etc. Cluster 5 (purple) is related to PARP inhibitors and contains 63 terms, including ATR, BRCA mutation, HRD, DNA-damage response, germline mutations, Niraparib, Olaparib, Rucaparib, Veliparib, RAD51, synthetic lethality, somatic/secondary mutations, etc. Cluster 6 (light blue) focuses on prognosis and contains 54 terms, including survival, impact, primary/interval debulking surgery, platinum-free interval, and secondary cytoreductive surgery, among others.

#### 3.5.2 Keywords burst detection and overlay visualization

Keyword burst detection generated by CiteSpace refers to detecting keywords with a high frequency of appearance in a certain period of time, which helps researchers analyze the evolution of PROC research as well as highlight emerging topics. The PARP inhibitor (strength, 11.81; time span, 2016–2022), homologous recombination (strength, 7.53; time span, 2016–2022), maintenance therapy (strength, 16.04; time span, 2017–2022), and neoadjuvant chemotherapy (strength, 12.32; time span, 2017–2022) are the research hotspots in the past decade, as is shown in **Figure 7C**. These words will likely continue in the near future. We also conducted the overlay visualization of keyword’s co-citation network time map (1990–2022) through CiteSpace to describe the evolution of key words in detail (**Figure 7D**).

## 4 Discussions

According to our knowledge, this is the first bibliometric analysis of PROC’s intellectual foundation and research fronts. In this study, bibliometrics and visual analysis were utilized to examine the global research trends of PROC research in terms of publications, contributing countries, institutions, journals, and keywords. Through keyword searches, 3,462 Web of Science publications from 1990 to 2022 were retrieved. These articles appeared in 671 scholarly journals and were written by 1135 authors from 75 countries and regions.

### 4.1 General information

The two studies that were originally used to define “platinum resistance” determined disease recurrence based on clinical symptoms, clinically detectable disease, and/or radiological evidence of disease recurrence (32, 33). During the first two years following the official coinage of the term “PROC,” only 16 articles were published. The number of publications per year is a crucial indicator of PROC’s strength and growth over a specific time period (19, 34). The annual output increased slowly but steadily prior to 2013 and exhibited a general upward trend. Afterwards, with the highest citation frequency, the “AURELIA” study (involving chemotherapy combined with bevacizumab) (8) in 2014 accelerated the annual output with the highest citation frequency. Their study was regarded as an important clinical study and a major advancement in PROC studies, with a very high frequency of citations. In addition, roughly 70 percent of all the literature extracted was published between 2014 and the present, demonstrating that PROC has garnered an increasing amount of academic interest in recent years.

Only six of the 75 countries and regions have published more than 200 papers, and it is worth noting that one-third of the publications were studies by American researchers. For research institutions, the University of Texas MD Anderson Cancer Center, which is affiliated with the United States, is the institution with the most publications and attention. Thus, the United States contributed significantly to the academic impact and renown of PROC research. In addition, China was the only developing nation to rank among the top five most productive nations. China is currently the second-most productive nation in PROC research, second only to the United States. Surprisingly, despite having fewer publications than other countries, Germany had the closest cooperation with other nations and the highest centrality. There is international cooperation regarding platinum-resistant OC, indicating that platinum-resistant OC is a global challenge.

As shown in **Tables 1** and **2**, the journals of Gynecologic Oncology and Journal of Clinical Oncology published the most papers about PROC research and received the largest number of co-citations, respectively. Most of the studies related to PROC research belong to the subjects of molecular biology, immunology, medicine, and clinical journals, which is consistent with the dual-map analysis (**Figure 5**). The dual-map overlay of academic journals represents their subject distribution (35). Evidently, the two major citation paths from Molecular/Biology/Immunology co-cited journals and Medicine/Medical and Clinical co-cited journals lead to Molecular/Biology/Genetics journals, indicating that PROC-related studies are currently focused on both basic and clinical research, such as the mechanism of platinum resistance, tumor immune microenvironment, genomic and epigenetic alterations, immunotherapy and targeted therapy, etc. The two main citation paths from Molecular/Biology/Immunology co-cited journals and Medicine/Medical and Clinical co-cited journals lead to Molecular/Biology/Genetics journals, implying that PROC-related studies are focused on both basic and clinical research nowadays, such as the mechanism of platinum resistance, the tumor immune microenvironment, genomic and epigenetic alterations, immunotherapy and targeted therapy, etc.

### 4.2 Knowledge base

Co-citation analysis can aid researchers in locating the common knowledge base shared by multiple studies in an efficient and convenient manner (36). In this bibliometric study, the top 10 co-cited references from the included PROC literature and the co-citation cluster network were as follows: **Table 3** and **Figure 6A**. We will investigate the PROC knowledge base based on the top 10 co-cited references, describing it in terms of both basic research and clinical trials. Despite a better understanding of the complex interplay between clonal selection and the microenvironment of resistant tumors, there is still much to learn about the complex and multiple mechanisms of platinum resistance (37). The Cancer Genome Atlas project analyzed messenger RNA expression, microRNA expression, promoter methylation, and DNA copy number in 489 high-grade serous ovarian adenocarcinomas and the DNA sequences of exons from coding genes in 316 of these tumors to better understand the molecular mechanism of platinum-resistance and the drivers of clinical phenotypes (27). On this basis, 92 patients with primary refractory, resistant, sensitive, and matched acquired resistant disease underwent whole-genome sequencing of tumor and germline DNA sample (38). In addition, the study revealed that CCNE1 amplification was prevalent in primary resistant and refractory diseases. Moreover, a number of studies have demonstrated that gene breakage frequently inactivates the tumor suppressors RB1, NF1, RAD51B, and PTEN in HGSC, and that multiple molecular events, including multiple independent reversions of germline BRCA1 or BRCA2 mutations in individual patients and loss of BRCA1 promoter methylation, contribute to acquired chemotherapy resistance (39, 40). Clearly, each of these studies laid the groundwork for subsequent molecular mechanism research. The prognosis for platinum-resistant ovarian cancer is bleak. In the past, Phase 2 results for targeted therapies, with the exception of bevacizumab, have been disappointing, with a 10% response rate in most studies (41–45). “AURELIA” was the most cited study in 2014 (NCT00976911) (8). This was the first randomized phase III trial combining bevacizumab with chemotherapy in platinum-resistant OC. The addition of bevacizumab nearly doubled progression-free survival (PFS) (3.4 *vs*. 6.7 months, HR 0.48, p = 0.001), but there was no improvement in overall survival (13.3 *vs*. 16.6 months, HR 0.85, p = 0.171). However, with single-agent chemotherapy and bevacizumab, the median survival time does not exceed 16 months. The need for alternative therapeutic options is urgent. As demonstrated by subsequent clinical studies (46–51), the emergence of PARP inhibitors in 2016 has brought hope to ovarian cancer patients (46).

### 4.3 Hotspot evolution and emerging topics

In bibliometrics, keyword co-occurrence (**Figures 7A, B**) can reflect the hotspots of an academic field, and the timeline view of keyword clusters (**Figure 7C**) can illustrate the emergence of new hotspots (52, 53). The high-frequency keywords of PROC research (**Figures 7A, B**) included chemotherapy, expression, apoptosis, activation, tumor microenvironment, PARP inhibitors (Olaparib, niraparib, rucaparib), pazopanib, maintenance therapy, BRCA1/2, PD-L1 expression, etc., which were regarded as the hotspots in PROC research. As time goes on, emerging topics occur continuously (**Figure 7C**). Cross-resistance, second-line therapy, gynecologic-oncology-group, topotecan, paclitaxel, etc., were among the terms that gained popularity in the early stages. During the stage of stable growth, new terms contained more mechanisms for detection and focused more on new and improved treatments, such as DNA methylation (54, 55), bevacizumab (56, 57), fallopian tubes, molecular mechanisms, neoadjuvant chemotherapy, immunotherapy, biomarkers, antibody-drug conjugates (58), PD-1, autophagy, homologous recombination deficit (47), immune checkpoint inhibitors, etc. Moreover, the cluster of keywords could describe the internal knowledge structure and reveal the research frontier of the discipline (19). Cluster analysis showed six main clusters in the PROC field, including the molecular mechanism of platinum, the fundamental research of ovarian cancer, chemotherapy, targeted therapy, PARP inhibitors and their molecular mechanisms, and the prognosis of PROC, which represent the six main hotspots of PROC research to some extent.

Notably, since OC is immunogenic, there was optimism that immunotherapy, a breakthrough treatment for several cancers over the past decade, could alter the disease’s progression. To increase response rates to anti-PD1/PD-L1 by adding chemotherapy, anti-angiogenic agents, PARP inhibitors, DNA damage (cyclophosphamide and/or radiotherapy), or other immune checkpoint inhibitors, multiple therapeutic strategies have been investigated in recent years (CTLA-4, etc.) (56, 59–62), which may improve the outcome of PROC by exploring new combination methods and screening advantaged populations on the basis of biomarkers.

Of particular note, folate receptor alpha (FRα) has emerged as a candidate for molecularly targeted therapeutic approaches that exploit its differential distribution pattern as a novel avenue for therapeutic intervention in PROC (63, 64). Due to its differential expression and the ability of FRα to internalize large molecules, FRα is well suited for antibody-drug conjugate (ADC)- based therapeutic strategies that can couple the targeting and pharmacokinetic features of an antibody with the cancer-killing impact of a cytotoxic agent (65). Mirvetuximab soravtansine (MIRV) (formerly IMGN853) is an ADC composed of an antifolate receptor alpha monoclonal antibody, a cleavable linker, and the maytansinoid DM4 payload, a potent tubulin- targeting antimitotic agent (66). To date, clinical experience with MIRV in ovarian cancer has demonstrated promising antitumor activity and a tolerable safety profile consisting predominantly of low-grade and reversible gastrointestinal and ocular adverse events (67, 68). In addition, the ongoing phase III randomized trial (MIRASOL; NCT04209855) is comparing MIRV versus investigator’s choice chemotherapy in high- FRα–expressing PROC, which revealed that MIRV monotherapy elicited high ORRs, durable responses, and a tolerable safety profile in patients with high- FRα PROC (69). On the basis of this differentiated safety profile, MIRV is additionally being assessed in other ongoing trials for platinum-sensitive ovarian cancer as a monotherapy (PICCOLO; NCT05041257) and in combination with carboplatin (NCT05456685) and bevacizumab (GLORIOSA; NCT0544577). Customized treatment strategies with combinatorial partners are an additional avenue worthy of further investigation. Taken together, MIRV highlights the potential for becoming a biomarker-driven, standard-of-care option in PROC and offers exciting future management options for this difficult-to-treat patient population.

### 4.4 Limitations

This is the first bibliometric analysis of PROC-related publications, including a quantitative analysis of such publications, that summarizes the evolution of PROC and may point the way for future research. However, there are certain restrictions. First, the studies were retrieved exclusively from the Web of Science, which may have resulted in publication bias. Second, all information was extracted using bibliometric tools based on machine learning and natural language processing, which, as other bibliometric studies (70) have reported, may result in bias. However, compared to the most recent traditional reviews (37, 71, 72, 73), our findings are largely consistent while providing researchers with more objective data, knowledge, and insight.

## 5 Conclusions

This is the first study to systematically analyze PROC-related publications using bibliometric and knowledge map methods. furthermore, we analyzed data using CiteSpace, VosViewer, and bibliometric (an R-tool for comprehensive science mapping analysis), resulting in richer results from a variety of perspectives. Unlike traditional reviews, this study offers an original and objective perspective on PROC research. It will be necessary in the future to continue strengthening cooperation between nations and institutions. Currently, the most active frontiers are concentrated on a better understanding of the immunological landscape of PROC, screening the population that can benefit from immunotherapy, and the clinical application of immune checkpoint inhibitors, especially in conjunction with other therapeutic options (such as chemotherapy and targeted therapy). We believe this study’s findings will serve as useful references for future research.

## Author contributions

YD, PZ, TZ, and LZ acquired, analyzed, and discussed the data. YD and PZ drafted the manuscript. RY designed the research, acquired the clinical information, and revised the manuscript. All authors have contributed to the manuscript and approved the submitted version.
