# Supplementary material for: Characterization of global research trends and prospects on platinum-resistant ovarian cancer: a bibliometric analysis
Source: Front Oncol. 2023 Jun 5;13:1151871. doi: 10.3389/fonc.2023.1151871 (PMC10277726; doi:10.3389/fonc.2023.1151871)
Supplement: Supplementary file 1 [file Table_1.docx]

**Supplemental Table 1 retrieval strategy for platinum-resistant OC**

| #1 | ((((((((TS=(Ovarian Neoplasms)) OR TS=(Neoplasm, Ovarian)) OR TS=(Ovarian Neoplasm)) OR TS=(Neoplasm, Ovary)) OR TS=(Ovary Neoplasm)) OR TS=(Ovary Cancer)) OR TS=(Cancer, Ovary)) OR TS=(Ovarian Cancer)) OR TS=(Cancer of Ovary) |
| --- | --- |
| #2 | (((((((((((TS=(Carcinoma, Ovarian Epithelial)) OR TS=(Carcinomas, Ovarian Epithelial)) OR TS=(Epithelial Carcinoma, Ovarian)) OR TS=(Ovarian Epithelial Carcinomas)) OR TS=(Epithelial Ovarian Cancer)) OR TS=(Ovarian Epithelial Cancer)) OR TS=(Ovarian Epithelial Cancers)) OR TS=(Cancer, Epithelial Ovarian)) OR TS=(Cancer, Epithelial Ovarian)) OR TS=(Carcinoma, Epithelial Ovarian)) OR TS=(Epithelial Ovarian Carcinomas)) OR TS=(Ovarian Carcinoma, Epithelial) |
| #3 | #1 OR #2 |
| #4 | (TS=(platinum-resistant)) OR TS=(platinum resistance) |
| #5 | #3 AND #4 |

Strategy A: #1 OR #2 Strategy B: #4 Strategy C: #3 AND #4.
